# The influence of psychological distance and topic type on inter-brain synchronization of emotion perception during face-to-face communication: an fNIRS hyperscanning study

**DOI:** 10.3389/fnins.2025.1670193

**Published:** 2025-10-08

**Authors:** Yun Tao, Meng Zhou, Jiayin Wang, Xuzhou Li, Xie Ma

**Affiliations:** ^1^Faculty of Education, Yunnan Normal University, Kunming, China; ^2^Key Laboratory of Educational Informatization for Nationalities (YNNU), Ministry of Education, Kunming, China

**Keywords:** psychological distance, sharing emotion, negative emotion, emotional perception, hyperscanning, fNIRS

## Abstract

Emotional perception plays a crucial role in social interaction; however, previous studies have majorly focused on static emotion perception rather than examining how emotions unfold during communication. Therefore, this study investigated how psychological distance (friends vs. stranger pairs) and topic type (shared vs. exclusive experiences) modulate inter-brain synchronization (IBS) during emotional communication, using functional near-infrared spectroscopy (fNIRS). The results showed that: (1) shared story (vs. exclusive story) elicited higher levels of emotion perception, both for self and other (*p* < 0.05), and friends (vs. stranger) perceived their friends’ negative emotions more strongly (*p* < 0.05). (2) Higher IBS was observed at the right superior frontal gyrus (rSFG; BA 10) when shared story in friend than shared story in stranger (*p__FWE_* < 0.05). The results indicate that with the deepening of friendships, the overlap between the two parties increases, resulting in higher emotional resonance when sharing common experiences. These findings provide brain imaging evidence supporting the involvement of emotion perception during communication.

## 1 Introduction

Emotion perception facilitates effective communication and cooperation. For instance, previous research has shown that emotional perception is beneficial not only for improving interpersonal relationships but also for enhancing group cohesion (Shamay-Tsoory, 2011; Li et al., 2022). Extensive studies have shown that social factors such as social relationships, spatial distance, and non-verbal behaviors play a critical role in emotion perception (Bucchioni et al., 2015). Notably, recent studies have begun to apply these findings in ecologically valid, real-world situations, such as emotional sharing, interpersonal touch, and communication involving conflict-related topics (Wang X. et al., 2022; Long et al., 2022; Lin et al., 2025). Although existing studies have identified neurocognitive correlates of emotion perception, the combined influence of psychological distance and topic characteristics on this process during face-to-face communication remains unclear.

Early investigations of emotion perception primarily used passive, unidirectional paradigms—including emotional face viewing (Bucchioni et al., 2015), film observation (Morgenroth et al., 2023), and auditory emotion processing (Koelsch et al., 2018). A representative finding reveals that observing loved ones’ painful stimuli elicits stronger activation of the anterior cingulate cortex and insula than observing strangers’ stimuli (Cheng et al., 2010). These studies consistently highlight the role of the psychological distance in emotion perception (Bucchioni et al., 2015; Morgenroth et al., 2023; Shao et al., 2024). According to the self-other overlap theory (Aron et al., 1991, 1992), interpersonal engagement enhances perceived closeness, thereby promoting behavioral, affective, and cognitive alignment (Anderson et al., 2003). Notably, heightened self-other overlap correlates with positive emotional states, shared goal representation, and enhanced mentalizing capacity (Waugh and Fredrickson, 2006; Myers et al., 2014; Tan et al., 2015). Similarly, neuroscientific evidence further corroborates these behavioral findings. Furthermore, dyadic neuroimaging studies demonstrate that friendship predicts interpersonal neural synchronization during joint movie viewing (Parkinson et al., 2018), along with structural convergence in social cognition networks, including the dorsomedial prefrontal cortex (dmPFC), temporoparietal junction (TPJ), superior temporal gyrus (STG), and amygdala (D’Onofrio et al., 2022). The TPJ has been consistently implicated in the self-other distinction and theory of mind (ToM). A large number of functional magnetic resonance imaging (fMRI) studies have reported TPJ activation while making judgments involving others, highlighting its central role in mentalizing (Ahmad et al., 2021; Long et al., 2023; Cheng et al., 2024).

Communication serves as a primary channel for emotional exchange, requiring simultaneous processing of semantic content (e.g., verbal messages) and paralinguistic features (e.g., prosody, facial expressions, and gestures) (Wang X. et al., 2022). Neuroimaging studies using dyadic fMRI paradigms reveal that speaker–listener interactions elicit interpersonal neural synchronization in language-related regions, including the inferior frontal gyrus (IFG), superior temporal gyrus (STG), and angular gyrus (AG). Furthermore, emotional valence enhances this synchronization within limbic regions such as the amygdala, hippocampus, and temporal pole (Smirnov et al., 2019; Ross et al., 2022). Recent work extends these findings to face-to-face communication, demonstrating that superior/middle frontal gyrus (SFG/MFG) synchronization encodes both emotional content and linguistic structure (Cheng et al., 2024; Carollo et al., 2025). However, few studies have investigated the role of different topic types (exclusive vs. shared) in communication. Although the perception of both exclusive and shared messages may be amplified when communicated to others, they may rely on different underlying mechanisms. Self-reference effect shows more processing bias toward self-related concepts (Rogers et al., 1977; Symons and Johnson, 1997; Zhao et al., 2015), and the medial prefrontal cortex (mPFC) plays a crucial role in processing these concepts (Yamawaki et al., 2017; Stendardi et al., 2021). Similarly, sharing personal experiences with another person can amplify these experiences (Boothby et al., 2014), with mirror neurons (MN) playing an important role in this process (Schmidt et al., 2021). The MN is a trimodal system composed of a neuronal population that responds to motor, visual, and auditory inputs, such as when an action is performed, observed, heard, or read about (Rizzolatti et al., 1996; Gallese et al., 2004; D’Ausilio, 2007). It reflects an integration of motor, auditory, and visual information processing, involving action understanding and recognition, which are central to language learning. Such integration may also form the basis for language-related constructs such as ToM (Le Bel et al., 2009; Sadeghi et al., 2022). A growing number of studies also focus on the role of mirror neurons system (MNS) in social interaction abnormalities observed in mental disorders (Hamilton, 2013; Mier et al., 2014; Valizadeh et al., 2022). Overall, communication engages shared neural mechanisms that link language, emotion, and social understanding.

The fNIRS-based hyperscanning approach is suitable for investigating dynamic interactions among multiple brains, thereby exploring the neural basis of social interactions (Czeszumski et al., 2020; Zhao et al., 2024). Unlike conventional fNIRS (single-brain), which focuses on the neural responses of individuals in isolation, fNIRS-based hyperscanning enables the simultaneous measurement of neural activity between two or more interacting individuals. This enables researchers to capture inter-brain synchronization (IBS), which reflects the coupling and information exchange that occur uniquely during real-time interpersonal interactions (Cui et al., 2012). Unlike traditional fNIRS analysis (e.g., task-related activation or within-brain connectivity), hyperscanning emphasizes cross-brain measures such as wavelet coherence (WTC) or Granger causality (GC), directly capturing interpersonal coordination (Cui et al., 2012; Jiang et al., 2012; Long et al., 2023). This approach advances social neuroscience by revealing neurocognitive mechanisms during interpersonal interactions (Cui et al., 2012; Babiloni and Astolfi, 2014; Tan et al., 2023). Previous studies have found widespread IBS during interpersonal interactions, such as synchronized movement (Cui et al., 2012; Liu et al., 2021), interpersonal communication (Jiang et al., 2012), economic decision-making (Zhang et al., 2021), creative activities (Mayseless et al., 2019), teaching activities (Pan et al., 2020, 2021, 2023), and interpersonal conflicts (Yang et al., 2020). Furthermore, IBS reflects the cohesion, quality of interaction, and shared information between interacting individuals (Jiang et al., 2012).

Empathy is another core construct that underlies emotion perception and interpersonal communication. Conceptually, empathy involves both affective sharing and cognitive perspective-taking (Healey and Grossman, 2018; Gillissen et al., 2025), thereby facilitating prosocial behaviors and interpersonal coordination (Zhou et al., 2022; Smith, 2025). Neuroimaging studies indicate that affective empathy is primarily supported by the anterior cingulate cortex and insula, while cognitive empathy involves higher order social cognition regions, including the mPFC and TPJ (Cheng et al., 2010; Ahmad et al., 2021; Cheng et al., 2024). Importantly, recent dyadic neuroimaging evidence further indicates that empathic traits modulate IBS during social interactions, thereby predicting the quality of cooperation, the degree of emotional alignment, and the effectiveness of conflict resolution (Wang S. et al., 2022; Li et al., 2024). Thus, incorporating empathy into the current framework is crucial for a more precise understanding of neurocognitive mechanisms that support face-to-face emotional communication.

The study examines IBS between friends and strangers during the disclosure of shared negative narratives (shared stories) vs. exclusive negative narratives (exclusive stories). To test these issues, we designed an experiment that incorporated two critical factors: psychological distance (between-subjects: friend/stranger) and topic type (within-subject: shared/exclusive). During communication, the brain activity of both members of the pair was simultaneously recorded by fNIRS systems. The prefrontal cortex (PFC) and right temporoparietal junction (rTPJ) are key regions involved in social interactions and are closely linked to mentalization (ToM) and emotional processing (Long et al., 2023; Cheng et al., 2024; Pan et al., 2023). We hypothesized that friends would show stronger IBS than strangers, and that shared stories would show stronger emotion perception than exclusive stories.

## 2 Method

### 2.1 Participants

G*Power (Faul et al., 2007) was used to calculate the required sample size with *f* = 0.25, α = 0.01, *power* = 0.95, which indicated that 54 pairs were needed. A total of 65 pairs (130 participants) were recruited from Yunnan Normal University. However, nine pairs were excluded due to excessive bad channels (>4 channels, 10% of all channels), failure to comprehend the instructions, and inability to complete the experiment. The final sample consisted of 56 pairs for the next data analysis. According to their self-reports about psychological distance (Aron et al., 2004), pairs with scores above the mean of all pairs (4.196) were classified as the friend group (26 pairs, including 24 female–female pairs), whereas dyads with scores below the mean were classified as the stranger group (30 pairs, including 25 female–female pairs). In addition, the two groups showed a significant difference in psychological distance and no significant difference in empathy (Table 1).

**Table 1:** Demographic of the friend and stranger groups.

	Friend (*n* = 26)	Stranger (*n* = 30)	*t*/*χ*^2^ value	Cohen’s d
Age	20.37 ± 2.40	21.08 ± 2.69	−1.48	0.28
Sex (male)	24 (2)	25 (5)	0.37	–
Handedness (left)	45 (7)	50 (10)	0.04	–
Psychological distance	5.35 ± 1.31	3.20 ± 1.10	−15.55^***^	2.95
Empathy	48.56 ± 6.22	48.62 ± 5.43	0.08	0.02

^*^p < 0.05, ^**^p < 0.01, ^***^p < 0.001.

Each participant had normal or corrected-to-normal vision, had no history of mental illness or brain injury, and gave written informed consent before taking part in the experiment. All participants were informed that the potential benefit of the study was contributing the findings to an academic journal. The studies involving human participants were reviewed and approved by the Ethics Committee of Yunnan Normal University (YNNU202409020020). The patients/participants provided their written informed consent to participate in this study.

### 2.2 Assessment of the psychological distance

In the present study, we used Inclusion of Others in the Self (IOS) scale to measure the psychological distance (Aron et al., 2004). The single-item scale consists of seven images, each presenting two circles. The two circles show increasing overlap, ranging from no overlap in the first image to complete overlap in the seventh. The extent of the overlap is intuitively understood by respondents as representing the closeness of the relationship between the subjects presented in the two circles, for example, between the responder and the “Other” identified in the circle; a greater overlap stands for a closer relationship. The respondent is asked to indicate which of the seven pictures best represents the relationship with the “Other.”

### 2.3 Assessment of empathy

Empathy was measured by the interpersonal reactivity index (IRI) (Davis, 1980). Four components included in the IRI are perspective taking, fantasizing, empathic concern, and personal distress. A total score of 28 could be obtained for each of the four subscales, and a higher score in a subscale represents higher functioning in each aspect of empathy.

### 2.4 Experimental materials

To obtain participants’ real self-experiences, two pair members (A and B) were asked to write two outlines of his or her stories when he or she arrived at the laboratory and before beginning the fNIRS scanning (Cheng et al., 2024). One outline was about a negative story that both of them had experienced together (*shared story*), and the other was about a negative story that only the individual had experienced (without A or B) (*exclusive story*).

Some examples were given by the examiner before writing, such as, “Last year, when we were in math class, we formed a group and needed to complete a group assignment. However, our grades were so bad that we were scolded by the teacher. I felt very sad at that time …” (*shared story*) and “Last week, I attended a student union dinner. My friends were all having a great time chatting, but I did not feel like I belonged in the group. I even felt like they were trying to exclude me, which made me angry…” (*exclusive story*). Each participant was informed that each story would take ~ 2 min to share. Second, the two examiners would confirm the validity of the materials after writing.

### 2.5 Tasks and procedures

**Figure 1:**
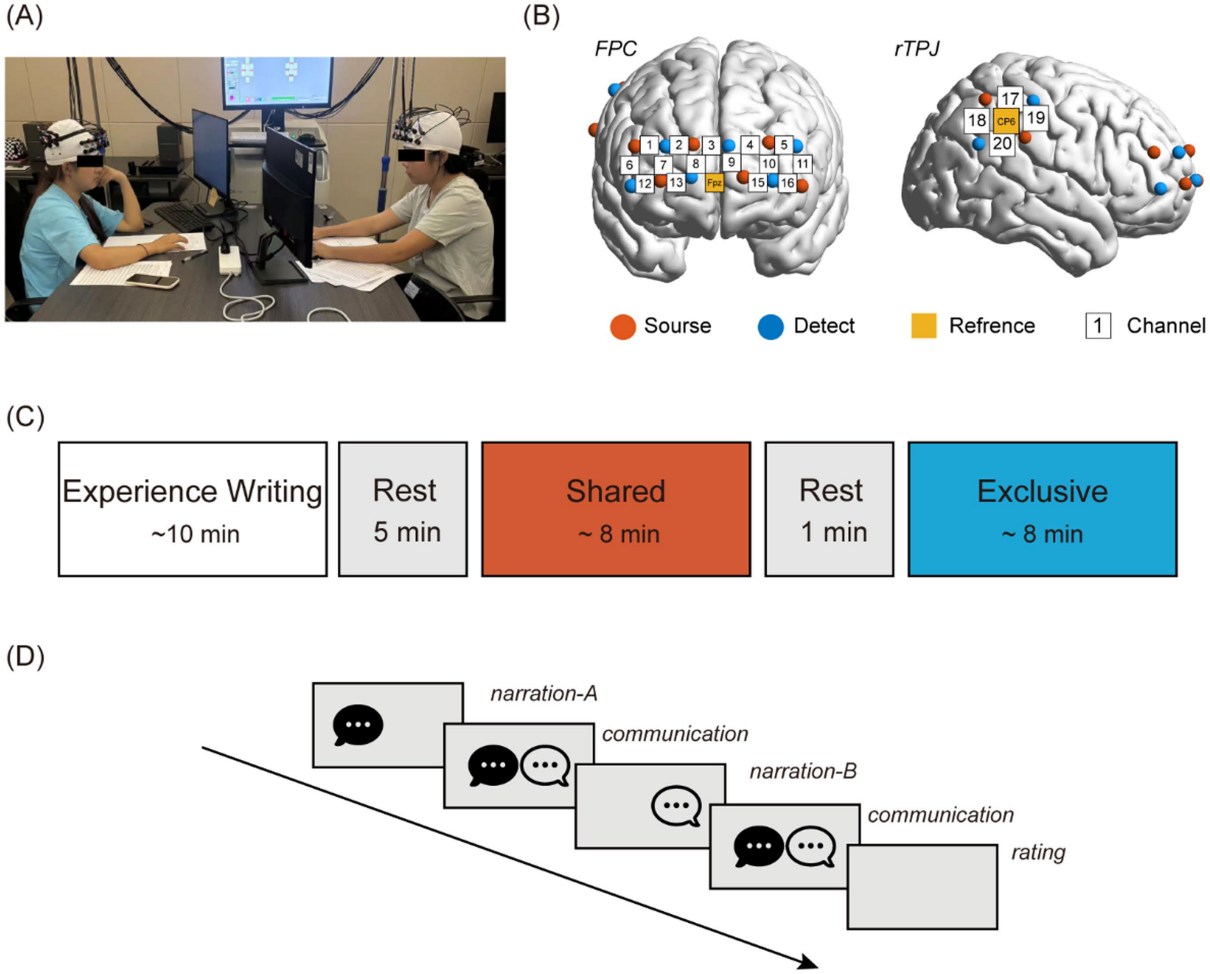
Experimental process and settings. **(A)** Experimental scene; **(B)** Probe set. Sixteen channels on the PFC and four channels on the rTPJ; and **(C)** experimental procedures. Before fNIRS scanning, participants were asked to write two outlines of their stories (shared and exclusive). Brain activity from the two participants was acquired simultaneously using fNIRS, including rest-state (5 min), and two talk sessions (shared and exclusive). **(D)** Example of one session: Each session consisted of two rounds. In the first round, Participant A shared a story while Participant B listened and communicated, followed by Participant B sharing a story while Participant A listened and communicated. After sharing stories with each other, they completed their behavioral assessment questionnaire (self-negative emotion perception and other-negative emotion perception).

The experiment was conducted in a silent room and consisted of four separate phases. First, the participants arrived at the laboratory in pairs. They first completed the experience writing task, then proceeded to the fNIRS task (Figure 1C).

Consistent with previous studies (Cheng et al., 2024), participants sat face-to-face during the task, whereas the experimenters left the room to provide a comfortable and private environment for the participants. The two pair members (A and B) were seated at a table facing each other at a distance of ∼90 cm (Figure 1A). Each pair of participants was required to complete a 5-min resting-state session, and to remain still with their eyes closed, relax their mind, and remain as motionless as possible (Cui et al., 2015; Lu et al., 2010).

Subsequently, participants engaged in two conditions in sequence: one for sharing shared stories and one for sharing exclusive stories. Each pair of participants was required to begin by completing the shared story before proceeding to the exclusive story. In each story, participants A and B took turns sharing stories (Figures 1C, D). A complete sharing process for one participant included a “narration” phase and a “communication” phase. In the narration phase, participant A (or B) narrated the experience to participant B (or A), who just listened without giving any verbal feedback (physical feedback was available as real talk). When participant A (or B) finished narrating, participants A and B freely communicated with each other about the experience. The two-phase sharing process lasted ~7–8 min. When both participants A and B had completed sharing their experiences (after approximately 15 min), the task session ended. Participants then filled out a behavioral assessment questionnaire, including self-negative emotion perception and other-negative emotion perception (5-point Likert score). In addition, participants received time and task (*exclusive* or *shared*) reminders delivered through a Psychtoolbox program (Brainard, 1997; http://psychtoolbox.org/). The program presented visual prompts (e.g., “Please ask A to describe the negative events that you have experienced together.”) and auditory prompts (“beep,” stop to talk). In addition, a voice recorder (ICD-PX470, Sony Co., Japan) was used to record conversations during the experiment.

### 2.6 Behavioral data analysis

We used R (version 4.4.1), dplyr package (version 1.1.4), and bruceR package (version 2024.6) for data analysis. To verify test for differences in ratings between shared and exclusive stories across the two groups, we conducted a 2 (psychological distance: friend, stranger) × 2 (topic type: shared, exclusive) repeated measures ANOVA (rmANOVA) on the subjective ratings of all participants, where psychological distance was the between-subjects variable and experience was the within-subjects variable.

### 2.7 fNIRS acquisition

The fNIRS signals of both paired members were recorded from the same 20 channels using an fNIRS system (LABNIRS, Shimadzu Co., Japan) at a sampling rate of 11.90 Hz. For each participant, two sets of optode probes covered two respective brain regions: the PFC was monitored with four emitters and four detectors, constituting 16 channels (denoted by “CH” and a number below); the rTPJ was monitored with two emitters and two detectors, forming four channels, following the international 10–20 system (see Figure 1B). The distance between the adjacent emitters and detectors was ∼3 cm, and the absorption of near-infrared light (wavelengths: 760, 803, and 850 nm). The precise positions of the fNIRS channels were measured by a 3D electromagnetic tracking device (FASTRAK; Polhemus, United States) and registered on the Montreal neurological Institute (MNI) brain space using a virtual registration method (see Table 2).

**Table 2:** Anatomical position for each recording channel.

Channel	MNI coordinates			Brain regions	
	*x*	*y*	*z*	AAL	BA
Prefrontal cortex					
CH-1	40	56	23	Frontal_Mid_R (0.979)	Dorsolateral prefrontal cortex (0.890)
CH-2	23	68	23	Frontal_Sup_R (0.730)	Frontopolar area (0.936)
CH-3	11	70	23	Frontal_Sup_Medial_R (0.652)	Frontopolar area (1.000)
CH-4	−15	68	24	Frontal_Sup_L (0.762)	Frontopolar area (0.989)
CH-5	−34	59	24	Frontal_Mid_L (0.788)	Dorsolateral prefrontal cortex (0.879)
CH-6	49	51	13	Frontal_Mid_R (0.9509)	Dorsolateral prefrontal cortex (0.800)
CH-7	33	66	13	Frontal_Sup_R (0.721)	Frontopolar area (0.923)
CH-8	16	72	15	Frontal_Sup_R (0.508)	Frontopolar area (1.000)
CH-9	−10	72	15	Frontal_Sup_L (0.559)	Frontopolar area (1.000)
CH-10	−27	67	15	Frontal_Sup_L (0.817)	Frontopolar area (0.932)
CH-11	−42	56	12	Frontal_Mid_L (0.918)	Dorsolateral prefrontal cortex (0.805)
CH-12	43	61	2	Frontal_Mid_R (0.622)	Frontopolar area (0.645)
CH-13	25	71	5	Frontal_Sup_R (0.878)	Frontopolar area (0.705)
CH-14	7	73	6	Frontal_Sup_Medial_R (0.661)	Frontopolar area (1.000)
CH-15	−18	72	6	Frontal_Sup_L (0.803)	Frontopolar area (0.877)
CH-16	−36	64	2	Frontal_Mid_L (0.373)	Frontopolar area (0.775)
rTPJ					
CH-17	59	−39	56	Parietal_Inf_R (0.738)	Supramarginal gyrus part of Wernicke’s area (0.768)
CH-18	60	−57	43	Parietal_Inf_R (0.619)	Angular gyrus, part of Wernicke’s area (0.537)
CH-19	67	−28	45	SupraMarginal_R (0.979)	Primary somatosensory cortex (0.398)
CH-20	68	−43	30	SupraMarginal_R (0.707)	Supramarginal gyrus part of Wernicke’s area (0.547)

Including Anatomical Automatic Labeling (AAL) and Brodmann Area (BA), measured by a 3D electromagnetic tracking device (FASTRAK; Polhemus, United States).

### 2.8 Preprocessing of fNIRS data

The fNIRS data were preprocessed with the Homer 2 toolbox (Huppert et al., 2009), based on MATLAB. In line with previous studies, the quality of fNIRS data was checked by visual inspection. All channels that did not show a clear heart band at around 1 Hz in the wavelet transform plot were identified as bad channels and were excluded from all further analysis (~10%). Subsequently, to further reduce possible artifacts, motion artifacts were identified, and corrected by a cubic spline interpolation method (Scholkmann et al., 2010). Subsequently, a bandpass filtering procedure (0.01–0.5 Hz) was performed to reduce noise. The remaining data were converted to oxyhemoglobin (HbO), which were calculated following the modified Beer–Lambert law. Since the HbO data is more sensitive to changes in cerebral blood flow than the HbR, this study only focused on HbO (Jiang et al., 2012; Pan et al., 2023).

### 2.9 Interbrain synchrony calculation

After preprocessing, we used wavelet transform coherence (WTC) analysis to estimate the neural synchrony between the analogous channels of each pair (Cui et al., 2012). The interbrain synchrony (IBS) were time-averaged across the rest and discussion periods and converted into Fisher z-values. Consistent with previous studies, we focused on the relative enhancement of IBS during the task phase compared to the rest phase. Thus, we subtracted the IBS of the resting phase from that of the task phase to obtain IBS increase (ΔIBS).

The following analyses were conducted to identify the differences in ΔIBS between the two tasks. First, we identified the task-relevant frequency of interest (FOI) by performing a series of one-sample t-tests on ΔIBS across all channels. The resulting *p*-values were determined by a statistically stringent threshold of *p* < 0.0005 (Zheng et al., 2018; Long et al., 2023). Based on this procedure, one FOI was obtained, and ΔIBS within this FOI was averaged (0.0204–0.0229 Hz, CH7–CH7). Repeated measures ANOVAs were performed on averaged ΔIBS of all channels in this band, and FWE correction was performed (*p* < 0.05). Followed by this, we used Pearson coefficient were calculated correlation between ΔIBS and emotion perception.

## 3 Results

### 3.1 Behavioral results

**Figure 2:**
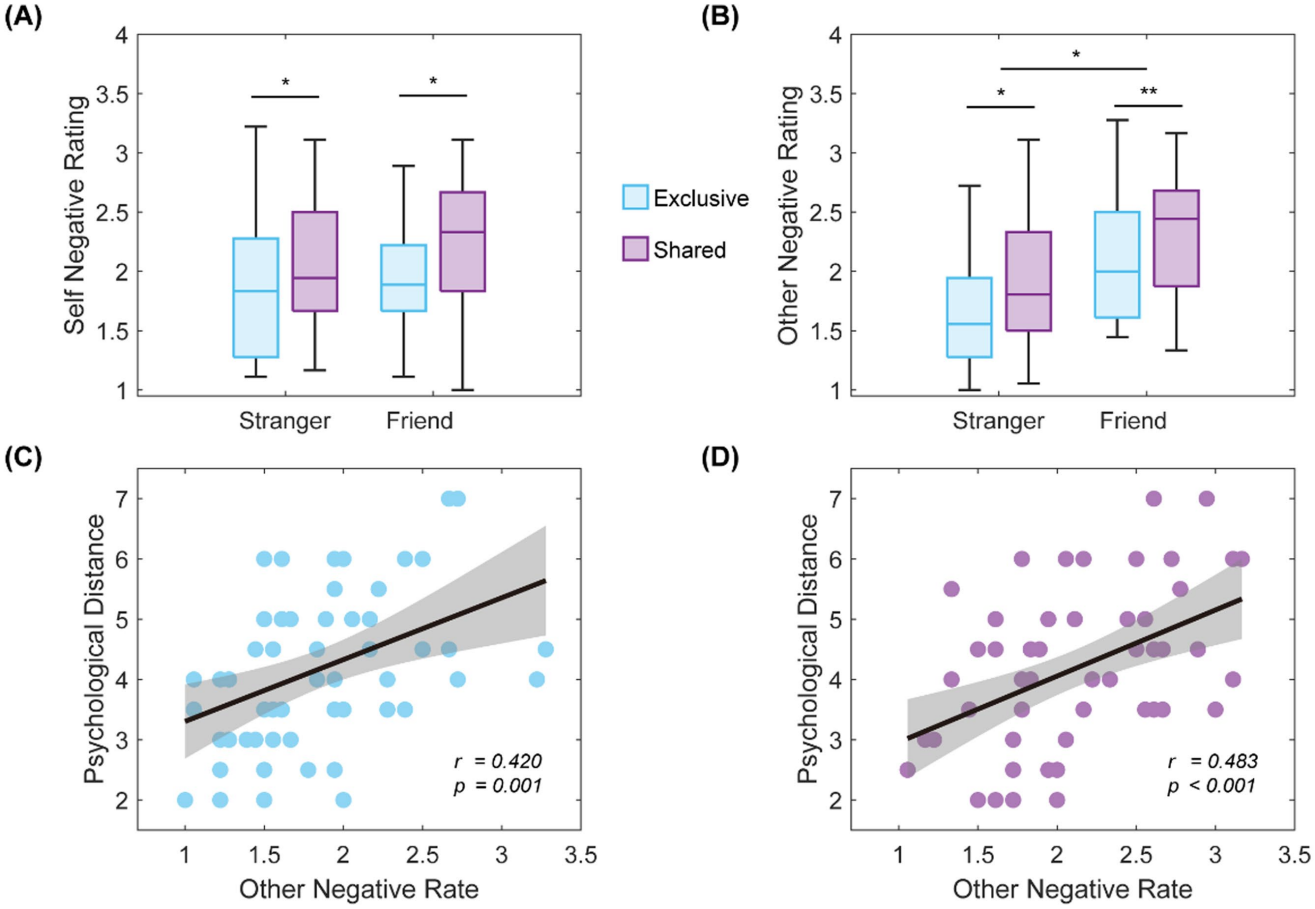
Behavioral statistical results graph. **(A)** Main effect of topic type on the negative; **(B)** Main effect of experience on topic type and psychological distance; **(C)** When sharing a shared story, the other-negative perception was positively correlated with psychological distance (*r* = 0.420, *p* = 0.001); **(D)** When sharing exclusive story, the other-negative perception was positively correlated with psychological distance (*r* = 0.483, *p* < 0.001). ^*^p < 0.05, ^**^p < 0.01, ^***^p < 0.001.

After 2 (psychological distance: friend, stranger) × 2 (topic type: shared, exclusive), rmANOVA analysis was performed on self-emotion perception. A significant main effect of the topic type was found [*F*(1, 54) = 9.82, *p* < 0.01, *η*2 *p* = 0.15], with *post-hoc* tests indicating that the self-emotion perception for shared story was higher than those for exclusive story (*p* < 0.05, Cohen’s d = 0.424, Figure 2A). Furthermore, when sex and empathy (mean scores between one pair) were included as covariates, an analysis of covariance (ANCOVA) results still showed a significant main effect of the topic type [*F*(1, 54) = 5.477, *p* = 0.021, *η*2 *p* = 0.048].

After 2 (psychological distance: friend, stranger) × 2 (topic type: shared, exclusive), rmANOVA analysis was performed on other-emotion perception. We found a significant main effect of the topic type [*F*(1, 53) = 11.06, *p* < 0.01, *η*2 *p* = 0.17] and groups [*F*(1, 53) = 9.50, *p* < 0.01, *η*2 *p* = 0.15], with *post-hoc* tests indicating that the other-emotion perception for exclusive story was higher than those for shared story (*p* < 0.05), and the other-emotion perception for friend was higher than stranger (*p* < 0.05, Figure 2B). Furthermore, when sex and empathy (mean scores between one pair) were included as covariates, ANCOVA results still showed a significant main effect of the topic type (*F*(1, 54) = 4.913, *p* = 0.029, *η*2 *p* = 0.044) and group (*F*(1, 54) = 8.938, *p* = 0.003, *η*2 *p* = 0.077).

Pearson’s correlation analysis between emotion perception and psychological distance showed that psychological distance positively correlated with other-emotion perception in both shared story (*r* = 0.42, *p* = 0.001, Figure 2C) and exclusive story (*r* = 0.48, *p* < 0.001, Figure 2D).

### 3.2 IBS results

**Figure 3:**
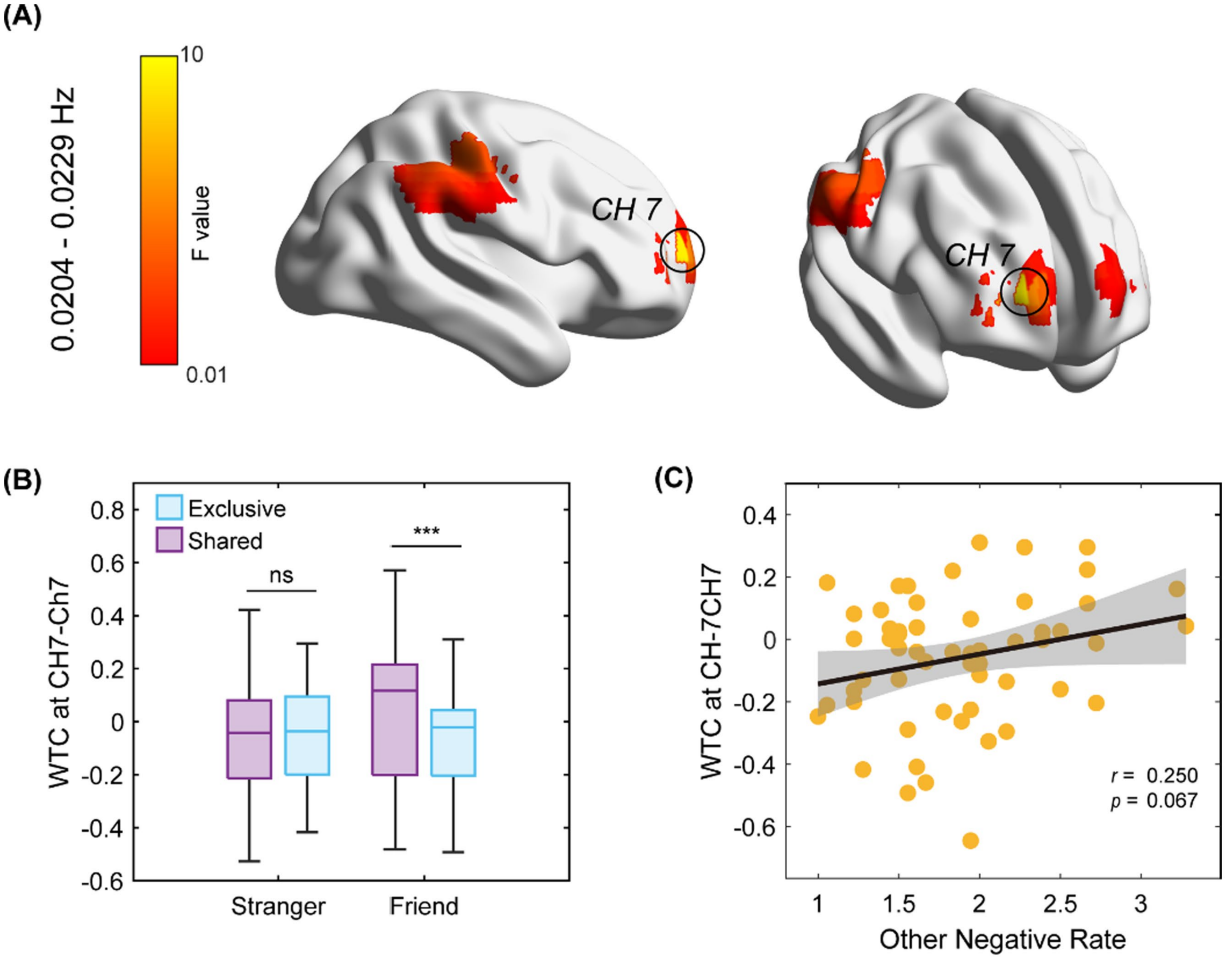
Statistical results of ΔIBS. **(A)** The location of significant interaction (group × topic type) of CH combinations on the cerebral cortex (CH 7 is BA 10); **(B)** Comparisons of ΔIBS at the frontopolar (CH 7–CH 7); **(C)** ΔIBS at the frontopolar (CH 7–CH 7) was marginally correlated with the other-emotion perception, when sharing exclusive experience. The gray area indicates the 95% confidence interval. Note: ^*^p__FWE_ < 0.05, ^**^p__FWE_ < 0.01, ^***^p__FWE_ < 0.001.

Within the FOI, rmANOVAs were performed on ΔIBS for each channel. The results revealed that ΔIBS at left frontopolar area (BA 10, CH 7) demonstrated significant interaction effects [*F*(1, 54) = 10.182, *p_no corr_* = 0.0024, *p_FWE_* = 0.005, *η*2 *p* = 0.159]. *Post-hoc* tests revealed that ΔIBS elicited by shared (compared to exclusive) story was significant in the friend’s group. Furthermore, when sex and empathy (mean scores between one pair) were included as covariates, ANCOVA results still showed a significant interaction [*F*(1, 52) = 10.891, *p* = 0.002, *η*2 *p* = 0.027]. Further analysis showed that ΔIBS at CH 7 was positively correlated with the perception of others’ negative emotions, when sharing exclusive experience (*r* = 0.25, *p* = 0.067) (Figure 3; Table 3).

**Table 3:** Results of rmANOVA on group × topic type.

Channel	Location	*F* (group)	*F* (group × topic type)	*F* (topic type)
1	DLPFC	0.650	0.707	1.376
2	Frontopolar area	0.492	1.274	2.088
3	Frontopolar area	0.020	1.156	1.218
4	Frontopolar area	2.147	0.089	0.683
5	DLPFC	0.041	0.097	0.605
6	DLPFC	1.353	0.023	0.054
***7***	***Frontopolar area***	***0.297***	***10.182*** ^**^	***0.008***
8	Frontopolar area	1.091	0.222	0.000
9	Frontopolar area	0.080	1.029	0.028
10	Frontopolar area	0.009	1.046	0.075
11	DLPFC	0.928	1.334	1.619
12	Frontopolar area	0.078	2.332	8.039
13	Frontopolar area	0.508	2.008	0.013
14	Frontopolar area	0.034	0.009	0.578
15	Frontopolar area	2.950	0.215	0.421
16	Frontopolar area	0.253	0.000	2.494
17	rTPJ	0.129	4.088	0.683
18	rTPJ	0.289	0.287	1.477
19	rTPJ	0.172	0.011	0.020
20	rTPJ	3.475	0.007	5.545

^*^p__FWE_ < 0.05, ^**^p__FWE_ < 0.01, ^***^p__FWE_ < 0.001. Results show main effect on the group, main effect on the topic type, and interaction effects (group × topic type), only significant at CH 7 (BA 10). Significant channels are highlighted in bold.

## 4 Discussion

In the present study, we used the fNIRS-based hyperscanning technique and nature communication paradigm to explore the effects of psychological distance and topic type on emotion perception in communication. We found that the shared story induced higher self-negative rates than the exclusive story, and friends rated higher other-negative than strangers. Moreover, the shared (vs. exclusive) story induced higher ΔIBS at the right superior frontal cortex (BA 10) in friends than strangers. These findings provide new insights into emotion perception during face-to-face communication.

### 4.1 Differences in emotion perception in the topic and group

The behavioral result showed that compared with exclusive story sharing, the shared story elicited enhanced perception of self-negative emotion. Firstly, the self-reference effect showed that people would pay more attention to information about self-relevant (Rogers et al., 1977). Compared with a shared story, an exclusive story does not involve the other person, and the experience mainly revolves around one’s own experience. Similarly, an experience with another person would amplify one’s experience (Boothby et al., 2014). This may reflect a social adaptation mechanism shaped by evolution. Group survival hinges on emotional sharing, a mechanism that drives emotional convergence among individuals. Such amplified emotional resonance facilitates information synchronization within the group, thereby optimizing coordinated collective action (Tomasello et al., 2012; Gallotti and Frith, 2013). Consequently, individuals attend more closely to the expression of their own feelings, which leads to stronger emotional perception.

Furthermore, result showed that the shared experience induced stronger negative emotion perception of others. Aligned with the self-other overlap theory, interpersonal engagement enhances perceived closeness, subsequently promoting behavioral, affective, and cognitive alignment (Anderson et al., 2003). The perceived intensity of pain is highest for close others (e.g., friends or romantic partners), followed by oneself, then strangers, and finally disliked individuals (Bucchioni et al., 2015). This differentiation in perception between in-group and out-group members serves as a critical adaptive function for individual survival (Niedenthal and Brauer, 2012). As a result, reduced psychological distance amplifies affective alignment through self-other neural coupling mechanisms.

Importantly, the behavioral results also showed that the friend’s group perceived the other person’s negative emotions more strongly than the stranger’s group. Previous studies showed that division of labor and collaborative cooperation were different forms of cooperation (Zhang et al., 2024), collaborative cooperation is more akin to sharing information than division of labor. Regardless of whether those participants were friends or romantic partners, the arousal level during conversations on conflicting topics was higher than during conversations on neutral topics (Long et al., 2022). Consistent with the self-other overlap theory, this phenomenon may be attributed to the merging of cognitive schemas that are formed through shared experiences.

To sum up, shared narratives would amplify dual emotional perception through self-referential focus and interpersonal synergy. Friends’ heightened sensitivity reflects evolved in-group prioritization via cognitive–emotional alignment, substantiating self-other overlap mechanisms.

### 4.2 IBS at BA 10 as a neural marker of behavior performance

The fNIRS results showed increased IBS elicited by shared (compared to exclusive) story, which was significant in the friend’s group at the right SFG. The frontal cortex plays a critical role in emotion recognition and information encoding (Abrams et al., 2011; Carollo et al., 2025), social interaction (Frith, 2007; Pinti et al., 2021), and ToM (Gilbert et al., 2006). In particular, the BA 10 was found to be involved in group communication (Nozawa et al., 2016) and collaborative task (Cui et al., 2012). Thus, our findings suggest that the right SFG (BA 10) might be engaged in processing the perception of others’ emotions during communication, and friends would be more capable of understanding each other’s emotions. Moreover, neural synchrony across individuals is seen as the alignment, reflecting shared attention or shared perspectives (Cui et al., 2012; Jiang et al., 2012; Long et al., 2022). Consistent with previous studies (Cheng et al., 2024), our findings suggest that enhanced IBS occurs in the shared story with friends, which might reflect a high level of cognitive alignment (e.g., joint attention and mutual understanding).

We did not observe negative perception, and IBS was moderated by psychological distance. Recent review involved that IBS transitions depend on specific context and goals, and plays an important role in interaction (Mayo and Shamay-Tsoory, 2024; Schilbach and Redcay, 2025). Therefore, the processing of exclusive and shared information differs. Specifically, heightened emotional perception of shared experiences does not necessarily entail increased IBS, whereas enhanced perception of individual experiences appears to require greater allocation of cognitive resources for processing and comprehension.

Moreover, IBS at the rTPJ was not observed. This result was not in line with our hypothesis, which predicted that the friend’s group would show stronger IBS during communication. Previous studies showed that the TPJ play an important role in interpersonal interaction (Van Overwalle, 2009; Park et al., 2021; Pan et al., 2023). The rTPJ represents the integration of prediction error signals, and greater prediction error was associated with increased rTPJ activity (Park et al., 2021). Therefore, two possibilities may account for these results: (1) shared experiences lead to the formation of collective memory, thereby enabling automated cognitive processing during subsequent discussions without requiring extensive involvement of mentalizing regions (Bargh et al., 2012). (2) The current experimental design’s lack of rigorous constraints on communication topics may be attributable to the heterogeneity in arousal levels across different subject matters. This critical dimension warrants systematic investigation in future research.

To sum up, fNIRS revealed friend-specific synchronization in the right superior frontal cortex (BA10) during shared storytelling, suggesting relational emotion coding via mentalizing–collaborative integration. Psychological distance influenced neural strategies: shared narratives relied on pre-aligned frameworks in close relationships, while exclusive stories required greater individual cognitive resource allocation, consistent with predictive coding models of social cognition.

### 4.3 Limitation

Despite the strengths of this study, it has several limitations. First, we only examined the activity at the PFC and rTPJ, whereas other key regions (such as the left TPJ, bilateral inferior frontal gyrus, and subcortical areas) are also critically involved in social cognition and the recognition of emotional prosody (Becker and Rojas, 2020; Leblanc and Ramirez, 2020; Gangopadhyay et al., 2021; Leipold et al., 2023). Thus, future studies should extend the coverage to include these relevant brain regions. Second, our study included nine male–male pairs and 39 female–female pairs, which may have introduced gender differences in empathy and IBS. Future research should further investigate gender-related variations in emotional contagion and their associated neural correlates (Rochat, 2023). Third, transcranial magnetic stimulation (TMS) and transcranial direct current stimulation (tDCS) could be used to experimentally test the causal relationships between psychological distance and IBS (Long et al., 2023). Lastly, all participants were recruited from China, which limits the generalizability of our findings across different cultural contexts. Future research should further investigate gender- and culture-related variations in emotional contagion and their associated neural correlates.

### 4.4 Conclusion

In summary, the present study tested the hypothesis that emotion perception of IBS differs between friends and strangers during different communication topics. The findings confirmed this hypothesis, demonstrating that psychological distance would influence other-negative feelings, and sharing (vs. exclusive) stories would induce stronger IBS in friends than in strangers. These findings provide new insights into the neurocognitive mechanism of emotion perception in communication across psychological distance.

## Data Availability

The raw data supporting the conclusions of this article will be made available by the authors, without undue reservation.
